# MDM2 Inhibits Axin-Induced p53 Activation Independently of its E3 Ligase Activity

**DOI:** 10.1371/journal.pone.0067529

**Published:** 2013-06-27

**Authors:** Ying He, Guili Lian, Shuyong Lin, Zhiyun Ye, Qinxi Li

**Affiliations:** State Key Laboratory of Cellular Stress Biology, School of Life Sciences, Xiamen University, Fujian, China; Innsbruck Medical University, Austria

## Abstract

MDM2 plays a crucial role in negatively regulating the functions of tumor suppressor p53. Here we show that MDM2 can inhibit Axin-stimulated p53-dependent apoptosis by suppressing p53 phosphorylation at Ser 46 and apoptosis-related p53 transactivational activity. Interestingly, the ubiquitin E3 ligase activity of MDM2 is not required for this inhibitory effect. Mechanically, either wildtype MDM2 or its E3-dead mutant, disrupts the Axin-based HIPK2/p53 complex formation by blocking the binding of p53 and HIPK2 to Axin. MDM2Δp53, a deletion mutant that lacks p53 binding domain fails to exert the inhibitory effect, demonstrating that the interaction of MDM2 and p53, but not its E3 ligase activity toward p53 plays key role in suppressing Axin-stimulated p53 activation. Our results thus have revealed a novel aspect of the mechanism by which MDM2 regulates p53 activities.

## Introduction

MDM2 (Mouse Double Minute 2) is an important negative regulator of the tumor suppressor p53. It interacts with and down-regulates p53 through several distinct modes including blocking p53 transactivational activity and promoting p53 degradation. It rigidly holds the cellular p53 protein level in check by virtue of its ubiquitin E3 ligase activity that targets p53 for degradation upon ubiquitination [1], [2]. The vital importance of MDM2 in down-regulating p53 was best demonstrated by a recent knock-in experiment in that mice harboring an MDM2 mutant deficient in E3 ligase activity died during early embryonic development unless these mice also lack p53 [3]. The homeostasis between MDM2 and p53 is achieved by a negative feedback loop: p53 activation leads to induction of MDM2 expression as *Mdm2* is a transcriptional target gene of p53, which in turn down-regulates p53 so that p53 is maintained at a lower level under normal condition [4]. Besides p53, MDM2 has also been shown to interact with many other proteins [4]. MDM2 can interact with and mediate the degradation of HIPK2 (Homeodomain-interacting protein kinase 2) which plays a crucial role in the phosphorylation of p53 at serine 46 following genotoxic stresses [5]. However, upon lethal DNA damages, HIPK2 can down-regulate MDM2 at posttranscriptional levels [6], indicating a close functional relationship between MDM2 and HIPK2.

Axin (Axis inhibitor) was first identified as a negative regulator of axis formation by acting as a key inhibitor of Wnt signaling [7]. Axin has now emerged as a master scaffold regulating p53 signaling and the activation of p53 in stress response [8]. In the case of p53 activation, we have shown that Axin interacts with and activates HIPK2 kinase to specifically phosphorylate p53 at Ser 46 [8]. Axin forms a p53 activating complex consisting of at least p53, HIPK2, and Daxx, in response to UV treatment. The importance of Axin is underscored by the observation that knockdown of *Axin* diminishes p53-dependent responses to genotoxic stress [9]. In the present study, we asked whether MDM2 plays a role in Axin-mediated p53 activation. We here show that MDM2 can inhibit Axin-induced p53 activation in different respects including p53 phosphorylation at Ser 46, p53 transactivational activity and p53-dependent apoptosis. Intriguingly, MDM2 inhibits Axin-induced p53 activation independently of its E3 ligase activity but through its ability to disrupt the Axin-based HIPK2/p53 complex formation.

## Materials and Methods

### Plasmid Construction

Full-length human MDM2 cDNA (GeneBank accession number: NM_002392) was obtained by amplifying cDNA of HEK 293 cells with primers: 5′-cgggatccatggtgaggagcaggcaaatg-3′ and 5′-ccgctcgagctaggggaaataagttag-3′, and cloned into *Bam*HI and *Xho*I sites of the mammalian expression vector pXJ with Myc or HA tagged at the N-terminus. MDM2 (C464A), MDM2ΔP53 and MDM2ΔRING were created by a PCR-based site-directed mutagenesis method (Stratagene). The cDNA fragments of MDM2 and its mutants were released from pXJ vectors with appropriate restriction enzymes, and then subcloned into pGEX-4T-1 vector to generate GST fusion proteins. The procedure for preparation of pCMV5-based Axin was described previously [10]. Breifly, the full-length Axin cDNA was obtained by screening a mouse pituitary λgt11 cDNA library using a polymerase chain reaction-generated 1-kilobase 5′-coding fragment as probe, tagged with HA, FLAG or Myc at the N-terminus, and cloned into the *Cla*I and *Bam*HI sites of the mammalian expression vector pCMV5. pCMV5-based plasmids for *p53* gene (GeneBank accession number: NM_000546) were obtained as a gift from Dr. V Yu (IMCB, Singapore). To construct His-tagged expression vectors for Axin and p53, full-length Axin cDNA released from CMV5-Axin with *Cla*I and *Sma*I was filled with Klenow and then cloned into *Eco*RV site of pET-32m vector, and full-length p53 cDNA released from CMV5-Myc-p53 with *Nde*I and *Sma*I was treated with Klenow and inserted into pET-32m vector digested with *Hin*dIII and blunted with Klenow. Full-length cDNA encoding HIPK2 was obtained by fusion of EST clones. *Axin* RNAi plasmid pSUPER-*Axin* was generated as described previously [8]. A pLL3.7-based siRNA with the sequence of GCCACAAATCTGATAGTAT was selected for specifically targeting to *Mdm2*.

### Cell Lines and Transient Transfection

HEK 293, HEK 293T and H1299 (NCI-H1299) cell lines were purchased from ATCC. U2OS cell line that had been originally purchased from ATCC was provided by Dr. V Yu (IMCB, Singapore) as a gift. All of these cell lines were maintained in DMEM medium, with 10% fetal bovine serum, 100 IU penicillin, and 100 µg/ml streptomycin. Transfections were performed in 60-mm dishes or 6-well plates using calcium phosphate precipitation method for HEK 293 and HEK 293T cells, and Lipofectamine 2000 (Invitrogen) for H1299 cells.

### Co-immunoprecipitation and Western Blotting

Antibodies used for immunoprecipitation and western blot include anti-HA (F-7), anti-Myc (9E10), anti-p53 (DO-1), anti-MDM2 (SMP14) antibodies (Santa Cruz Biotechnology Inc.), anti-FLAG M2 antibody (Sigma), anti-p53 phospho-Ser 46 (Cell Signaling Tech.) and homemade rabbit anti-Axin and anti-p53 antibodies. Cell lysates were prepared and immunoprecipitated, followed by western blotting as previously described [8].

### p53-luciferase Reporter Gene Assay

HEK 293 cells growing on 6-well plates were transfected with 0.5 µg of pCMV5-LacZ, 0.5 µg of p53-luciferase reporter (Stratagene), 2 µg HA-Axin, together with 4 µg of Myc-MDM2 or its mutants. The total amount of transfected DNA of each well was adjusted to 7 µg with the empty vector pCMV5 where necessary. At 24 h post-transfection, cells were lysed and measured for β-galactosidase and luciferase activities (Promega). The values of luciferase activities were normalized by β-galactosidase readings. Data are presented as means plus standard deviation from three separate experiments performed in triplicate.

### Apoptosis Assay

U2OS and H1299 cells were plated on glass coverslips in 6-well plates. Cells were transiently transfected with 0.5 µg pEGFP-C3 (Clontech), 2 µg HA-Axin, together with 4 µg of Myc-MDM2 or its mutants. At 24 h post-transfection, apoptosis assays were performed as previously described [8].

### In *vitro* Binding Assay

The proteins His-Axin, His-p53, GST-MDM2, GST-MDM2 (C464A) and GST- MDM2Δp53 were expressed in BL21 bacterial cells (purchased from Invitrogen) induced by 1 mM IPTG for 6 h at 26°C, then were purified using His-select nickel affinity gel (Sigma) or glutathione-agarose beads (GE). 1 µg of His-Axin, 1 µg of His-p53, 6 µg of GST-MDM2, GST-MDM2 (C464A) or GST-MDM2Δp53 were mixed in different combinations. Mixed proteins were incubated with rabbit anti-p53 antibody bound to protein A/G beads in lysis buffer for 3 h at 4°C [8]. Precipitated proteins were washed by lysis buffer for 3 times and detected by western blotting using the appropriate antibodies.

## Results

### MDM2 Abrogates Axin-induced p53 Activation Independently of E3 Ligase Activity

**Figure 1:**
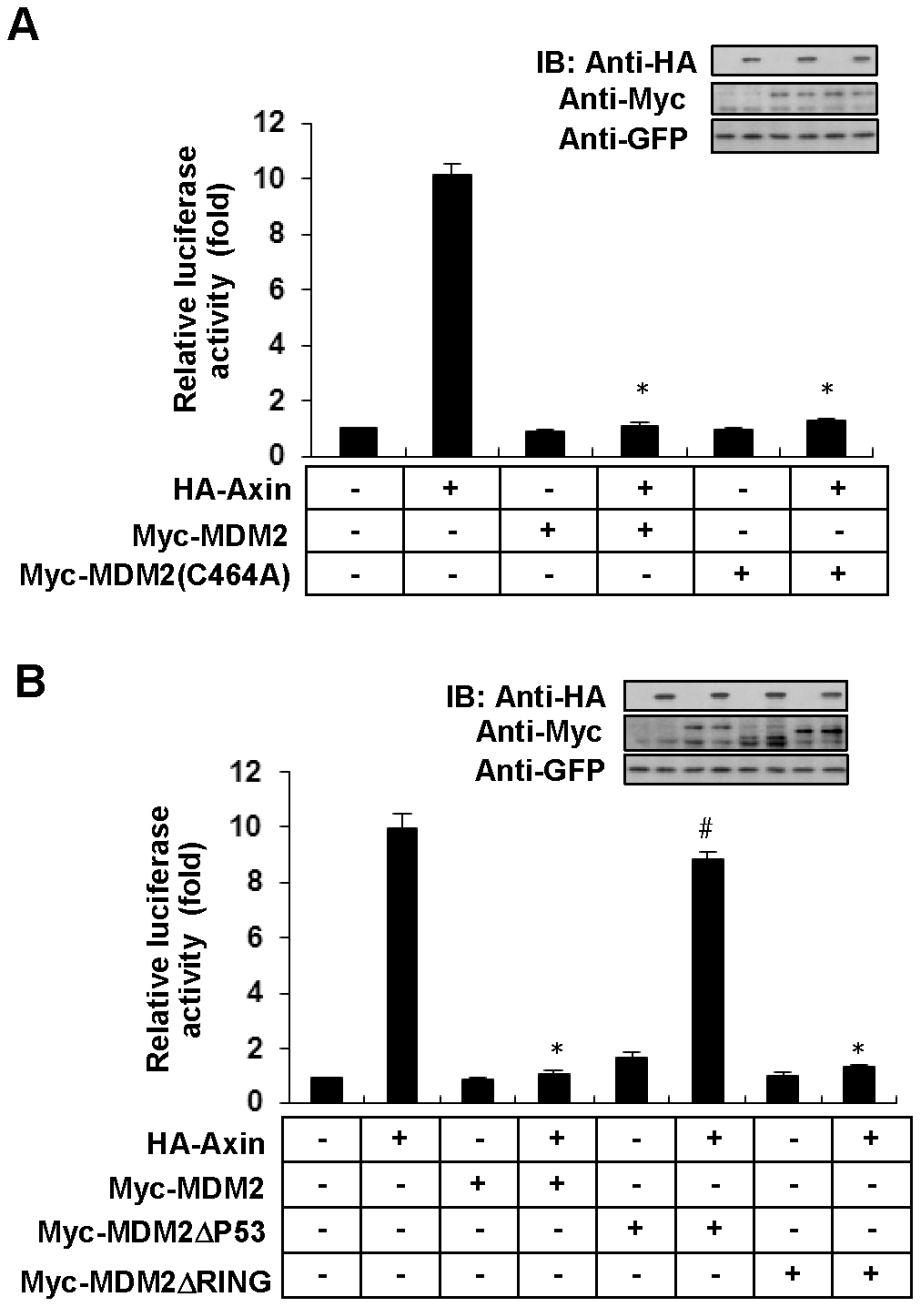
MDM2 and its E3-inactivated mutant MDM2(C464A) show the similar effect on inhibition of Axin-induced p53 transcriptional activity. (A) HEK 293 cells were transfected with p53-Luc reporter, HA-Axin, Myc tagged MDM2 and its mutants in different combinations as indicated. Western blotting were performed to indicate protein expression levels (inset). All transfections were performed in duplicate and the data are means±s.d. of three independent experiments. *, *p*<0.001 compared with cells transfected with HA-Axin alone (second column). Statistical analyses were done using *t* test. (B) Experiments were performed as in (A). *, *p*<0.001 compared with cells transfected with HA-Axin alone (second column); ^#^, *p*>0.05 compared with cells transfected with HA-Axin alone (second column).

As MDM2 is the crucially negative regulator of p53 activity identified hitherto and Axin is positive regulator of p53 activity. We want to know whether MDM2 inhibits Axin-induced p53 activation. To address this question, we generated MDM2-expressing vector from the cDNA of HEK 293 cells and detected the E3 activity of wild type MDM2 toward p53. As shown in Figure S1, wild type MDM2 showed strong E3 activity toward p53, in contrast, MDM2 (C464A), an E3 ligase-dead mutant of MDM2, completely lost E3 activity toward p53. Then we investigated the regulatory effect of MDM2 on Axin-stimulated p53 activation by using the PathDetect p53 cis-Reporting System (Stratagene) that carries the p53-specific enhancer elements [8], [11], [12]. As shown in Figure 1A, MDM2 can strongly decrease luciferase activation induced by Axin. We then asked whether ubiquitin E3 ligase activity of MDM2 is essential for its inhibitory effect on Axin-induced p53 transactivity and performed luciferase reporter assay by using E3 ligase-dead mutant, MDM2(C464A). Surprisingly, we found that MDM2(C464A) exhibited the same inhibition on Axin-induced p53 transcriptional activity as did wild type MDM2 (Figure 1A). Consistently, MDM2ΔRING, another E3 ligase-dead mutant of MDM2 that is deleted for its RING domain retained the ability of MDM2 on inhibition of p53 transactivity induced by Axin, indicating that E3 activity of MDM2 is not required to inhibit Axin-mediated p53 activation (Figure 1B). However MDM2Δp53, an MDM2 mutant lacking p53-binding domain, fails to exert the inhibitory effect, indicating that the inhibition may be based on the interaction between MDM2 and p53 (Figure 1B).

### MDM2 (C464A) Robustly Inhibits Axin-stimulated p53 Ser 46 Phosphorylation

**Figure 2:**
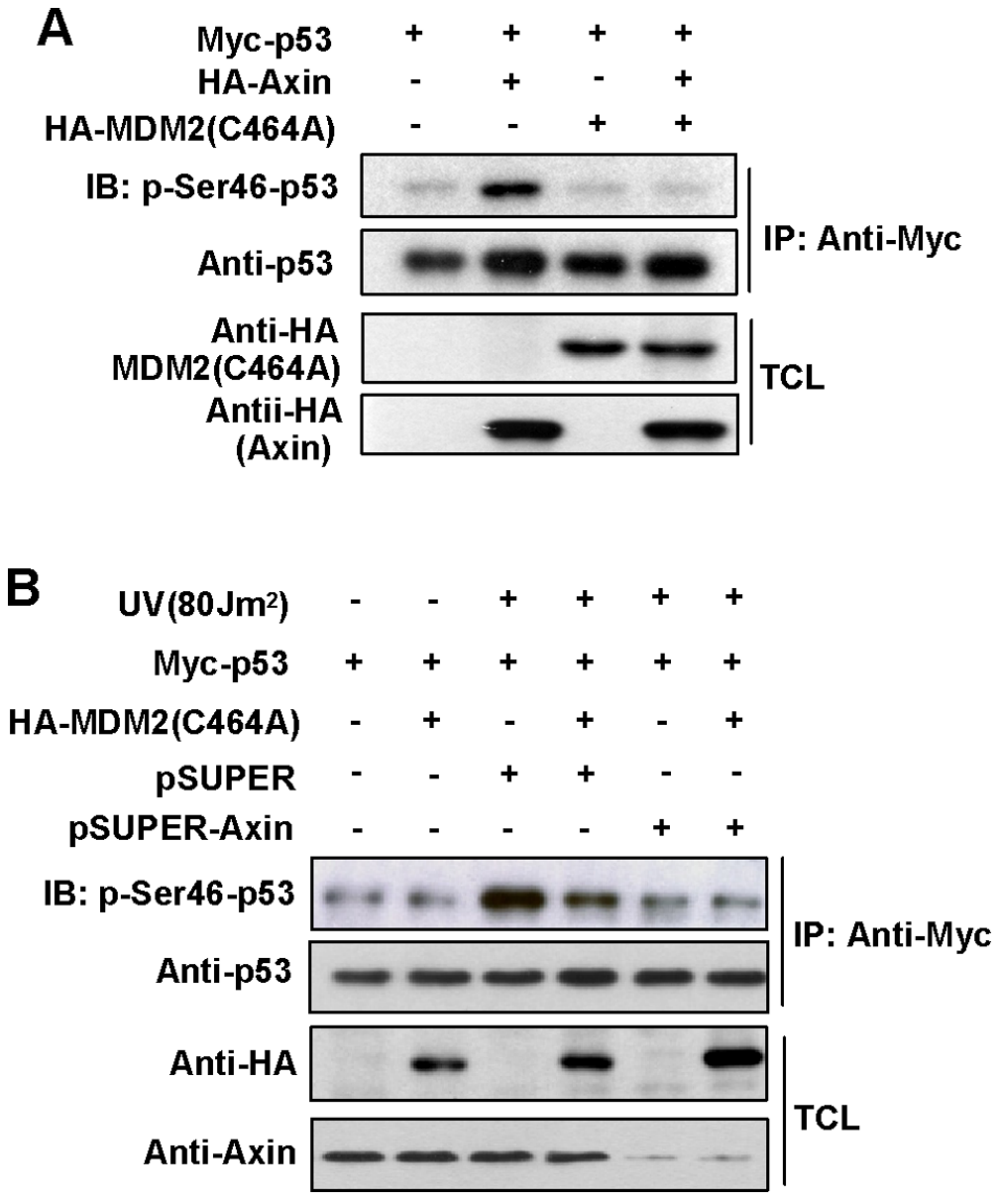
MDM2 (C464A) dramatically inhibits p53 Ser 46 phosphorylation. (A) H1299 cells were transfected with Myc-p53, HA-Axin or HA-MDM2 (C464A) as indicated, and analyzed by immunoprecipitation and western blotting. (B) H1299 cells were co-transfected with Myc-p53, MDM2 (C464A) and pSUPER-Axin in different combinations. 24 h after transfection, cells were treated with UV (ultraviolet) of 80 J/m^2^. At 6 h post-treatment, cells were lysed and immunoprecipitated, followed by western blotting with anti-p53 and anti-phospho-Ser 46 antibodies.

As Axin can stimulate p53 phosphorylation at Ser 46 by facilitating HIPK2 kinase activity [8], here we tested whether this effect of Axin can be blocked by MDM2. When p53 and MDM2 were co-overexpressed in H1299 cells, p53 is ubiquitinated and degraded leading to the basal level of p53 is lower than that in control cells transfected with Axin, p53 and blank vector (data not shown), which makes it difficult to compare the difference of p53 Ser 46 phosphorylation levels between cells overexpressed with and without MDM2. To avoid p53 degradation mediated by MDM2, MDM2 (C464A) was transfected together with Axin and p53. The result showed that Axin alone strongly activated p53 Ser 46 phosphorylation, while this effect was abrogated by co-expression of MDM2 (C464A) (Figure 2A). This observation was confirmed by another result showing that both overexpression of MDM2 (C464A) and knockdown of *Axin* can decrease UV-induced p53 Ser 46 phosphorylation to the same extent (Figure 2B), consistent with our previous work proved that Axin plays an important role in UV-induced p53 Ser 46 phosphorylation [8]. Taken together our results demonstrate that MDM2 can inhibit Axin-induced p53 phosphorylation at Ser 46 independently of it E3 ligase activity.

### MDM2 and MDM2 (C464A) Exhibit the same Inhibitory Effect on Axin-induced Apoptosis

**Figure 3:**
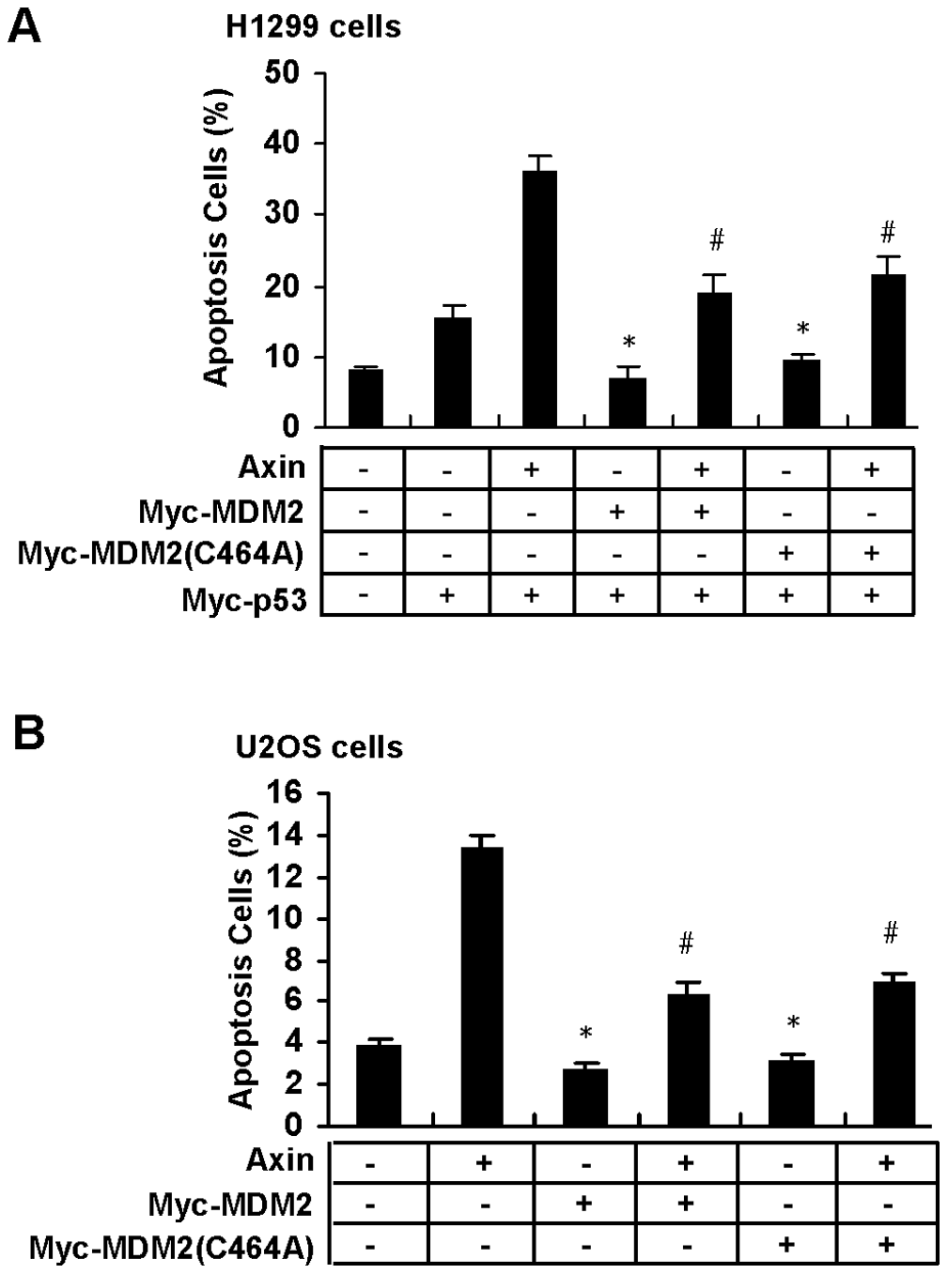
MDM2 and MDM2 (C464A) inhibit Axin-induced apoptosis to the same extent. (A) H1299 cells were transfected with GFP, Myc-p53, untagged Axin, Myc-MDM2 or Myc-MDM2 (C464A) in the combinations as indicated. Cell death was quantified 24 h after transfection by Hoechst 33324 staining and results were means±s.d. of three independent experiments. *, *p*<0.01 compared with cells transfected with p53 alone (second column); ^#^, *p*<0.01 compared with cells co-transfected with p53 and Axin (third column). Statistical analyses were done using *t* test. (B) U2OS cells were transfected with GFP, untagged Axin, Myc-MDM2 or Myc-MDM2 (C464A) in different combinations as indicated. The percentage of apoptotic cells was determined as in (A). *, *p*<0.05 compared with untransfected cells (first column); ^#^, *p*<0.01 compared with cells transfected with Axin (second column).

Overexpression of Axin can trigger cell to undergo apoptosis by stimulating p53 apoptosis-inducing function based on selective activation of *PUMA* transcription [9]. We want to know whether MDM2 can serve as an inhibitor on Axin-induced p53-dependent apoptosis. As indicated in Figure 3A, both MDM2 and MDM2 (C464A) can dramatically inhibit Axin-induced apoptosis in H1299 cells. Similar results were observed in U2OS cells (Figure 3B).

### Both MDM2 and its Mutant MDM2 (C464A) Prevent the Formation of Axin/p53/HIPK2 Comple*x*

**Figure 4:**
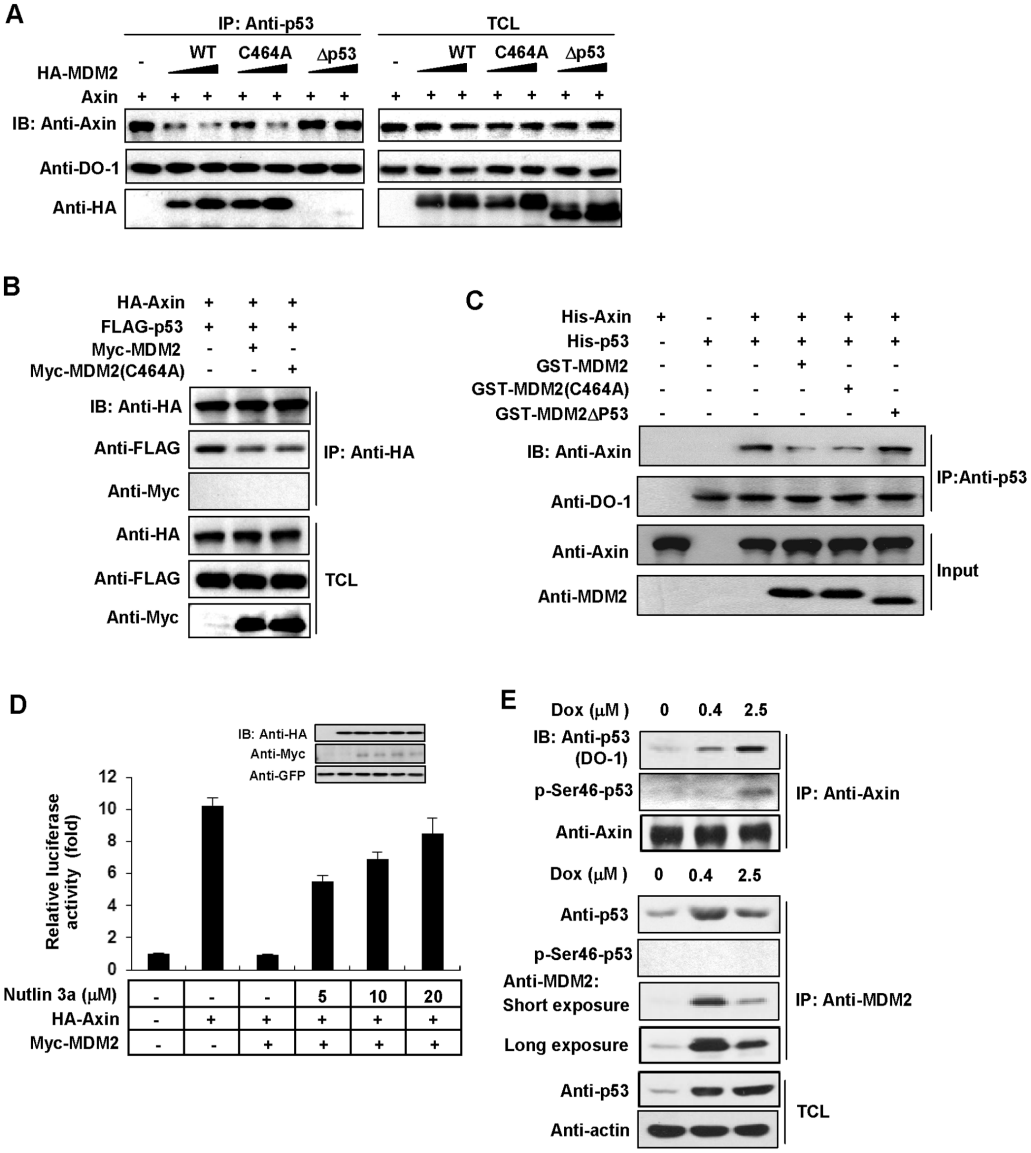
Both MDM2 and MDM2 (C464A) disrupt Axin-p53 interaction by recruiting p53. (A) HEK 293T cells were transfected with 2 µg untagged Axin and increasing amounts (3 µg and 8 µg) of HA-MDM2, HA-MDM2 (C464A) or HA-MDM2Δp53. (B) HEK 293T cells were transfected with HA-Axin, FLAG-p53 and Myc-MDM2 or MDM2 (C464A) and analyzed by immunoprecipitation and western blotting. (C) In normal lysis buffer, 1 µg purified His-Axin, 1 µg His-p53 and 6 µg GST-MDM2, GST-MDM2 (C464A) or GST-MDM2Δp53 were added and p53 was immunoprecipitated following by western blotting. BSA was used to make the protein amount of each sample equal. (D) HEK 293 cells were transfected with different combinations of pCMV5-LacZ, p53-luciferase reporter, HA-Axin, and Myc-MDM2 as indicated. At 6 h after transfection, cells were treated without or with 5 µM, 10 µM and 20 µM Nutlin 3a respectively for another 24 h followed by measure of luciferase activity. Results shown are mean±s.d. of three independent experiments. (E) U2OS cells were plated in 100 mm dishes at 70% confluence, followed by treatment with 0 µM, 0.4 µM and 2.5 µM doxorubicin respectively. 24 h after treatment, cells lysates were divided into two parts, one was immunoprecipitated with anti-Axin antibody, the other part was immunoprecipitated with anti-MDM2 antibody.

We next investigated the molecular mechanism by which MDM2 inhibits Axin-induced p53 activation. As Figure 1B indicated that this inhibitory effect of MDM2 may be based on its interaction with p53, we want to know whether MDM2 can compete with Axin for binding to p53. As expected, decreasing amounts of Axin immunoprecipitated with p53 were detected when increasing amounts of MDM2 or MDM2 (C464A) were overexpressed. It is important to note that E3 ligase dead MDM2 (C464A) showed the similar affinity with p53, consistent with the previous investigation [13]. In contrast, increasing amounts of MDM2Δp53 failed to interrupt Axin-p53 interaction (Figure 4A). This result was confirmed by a reciprocal immunoprecipitation assay showing that p53 precipitated with Axin was reduced by co-expression of MDM2 and MDM2 (C464A) in the same degree (Figure 4B). It is noteworthy that neither wildtype MDM2 nor MDM2 (C464A) showed any interaction with Axin, which excludes the possibility that MDM2 and Axin can bind to each other through their p53-binding sites and thus interferes the interaction of p53 with them (Figure 4B). In addition, we detected increased interaction between p53 and Axin in U2OS cells with endogenous *Mdm2* knocked-down by pLL3.7-based siRNA (Figure S2). More importantly, we performed an in *vitro* competitive assay by using purified proteins. As shown in Figure 4C, Axin in p53 immunoprecipitate was decreased by addition of GST-MDM2 or GST-MDM2 (C464A), but not by supplementation of GST-MDM2Δp53. Another evidence was that Nutlin 3a, a small molecular inhibitor of MDM2-p53 interaction [14], can neutralize the inhibitory effect of MDM2 on Axin-induced transcriptional activity of p53 (Figure 4D). We previously found that Axin can form distinct protein complexes in response to sublethal (0.4 µM) and lethal (2.5 µM) doses of doxorubicin treatment [15]. Then we investigated whether MDM2-p53 and Axin-p53 interactions can be affected by different dosages of doxorubicin treatment at endogenous protein levels. As shown in Figure 4E, upon sublethal treatment (lane 2), the protein level of MDM2 was highly increased, and the protein level of p53 co-immunoprecipitated with MDM2 was much more than that precipitated with Axin, indicating that under this condition, p53 is mainly occupied by MDM2 to avoid being sequestered and activated for apoptosis-inducing function by Axin. Upon lethal treatment (lane 3), the expression of MDM2 was robustly decreased. Consistently, the majority of p53 was captured by Axin complex. Interestingly, upon lethal treatment, high level Ser 46 phosphorylation was detected in Axin-occupied p53, but not in p53 binding to MDM2. These experiments indicated that both the competition between MDM2 and Axin for p53 interaction and the phosphorylation state of p53 occupied by MDM2 or Axin have great impacts on cells exposed to distinct doses of DNA damage.

### Both MDM2 and MDM2 (C464A) Inhibit Axin-HIPK2 Interaction

**Figure 5:**
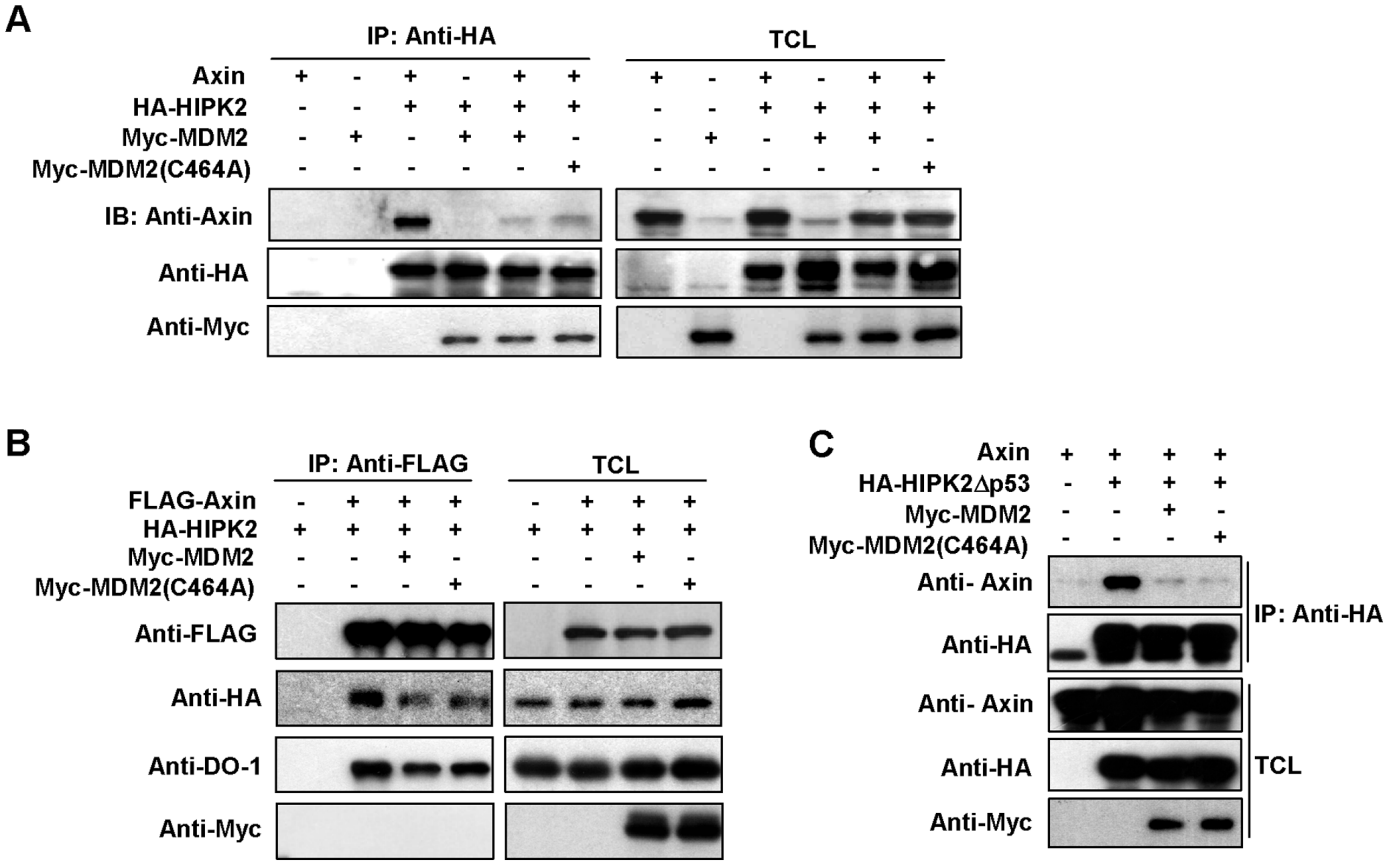
Both MDM2 and MDM2(C464A) inhibit Axin-HIPK2 interaction. (A, B) HEK 293T cells were co-transfected with Axin, HA-HIPK2 and Myc-MDM2 or MDM2 (C464A) as indicated, followed by immunoprecipitation and western blotting analysis. (C) Both MDM2 and MDM2 (C464A) can disrupt the interaction between HIPK2Δp53 (a HIPK2 deletion mutant that fails to bind with p53) and Axin. HEK 293T cells were co-transfected with different combinations of plasmids as indicated, followed by immunoprecipitation and western blotting.

Because MDM2 is another binding partner of HIPK2 [5], we investigated whether MDM2 can show any interference on the binding between Axin and HIPK2. As shown in Figure 5A, Axin precipitated by HIPK2 was drastically decreased by introduction of MDM2 or its mutant MDM2 (C464A), which demonstrates that both MDM2 and its E3-inactivated mutant MDM2 (C464A) can interrupt the interaction between Axin and HIPK2. Consistently, when Axin was immunoprecipitated, both HIPK2 and p53 in the precipitates were simultaneously reduced by co-expression of MDM2 or its mutant MDM2 (C464A) (Figure 5B). It is important to note that Axin-tethered p53 includes two pools, one directly interacts with Axin through the MID domain [8], the other associates indirectly with Axin using HIPK2 as a bridge. So MDM2 precipitated with HIPK2 may also contain two parts, one directly associates with HIPK2, the other binds indirectly to HIPK2 with p53 as a medium. To clarify which part of MDM2 plays key role in disrupting the interaction between HIPK2 and Axin, we generated HIPK2Δp53, a HIPK2 deletion mutant that fails to bind with p53 and hence is deprived of indirect interaction with MDM2. Immunoprecipitation assay showed that the interaction of this mutant with Axin could still be robustly inhibited by overexpression of either MDM2 or MDM2 (C464A), indicating that MDM2 disrupts HIPK2-Axin complex by directly binding to HIPK2 and releasing Axin from it (Figure 5C).

## Discussion

**Figure 6:**
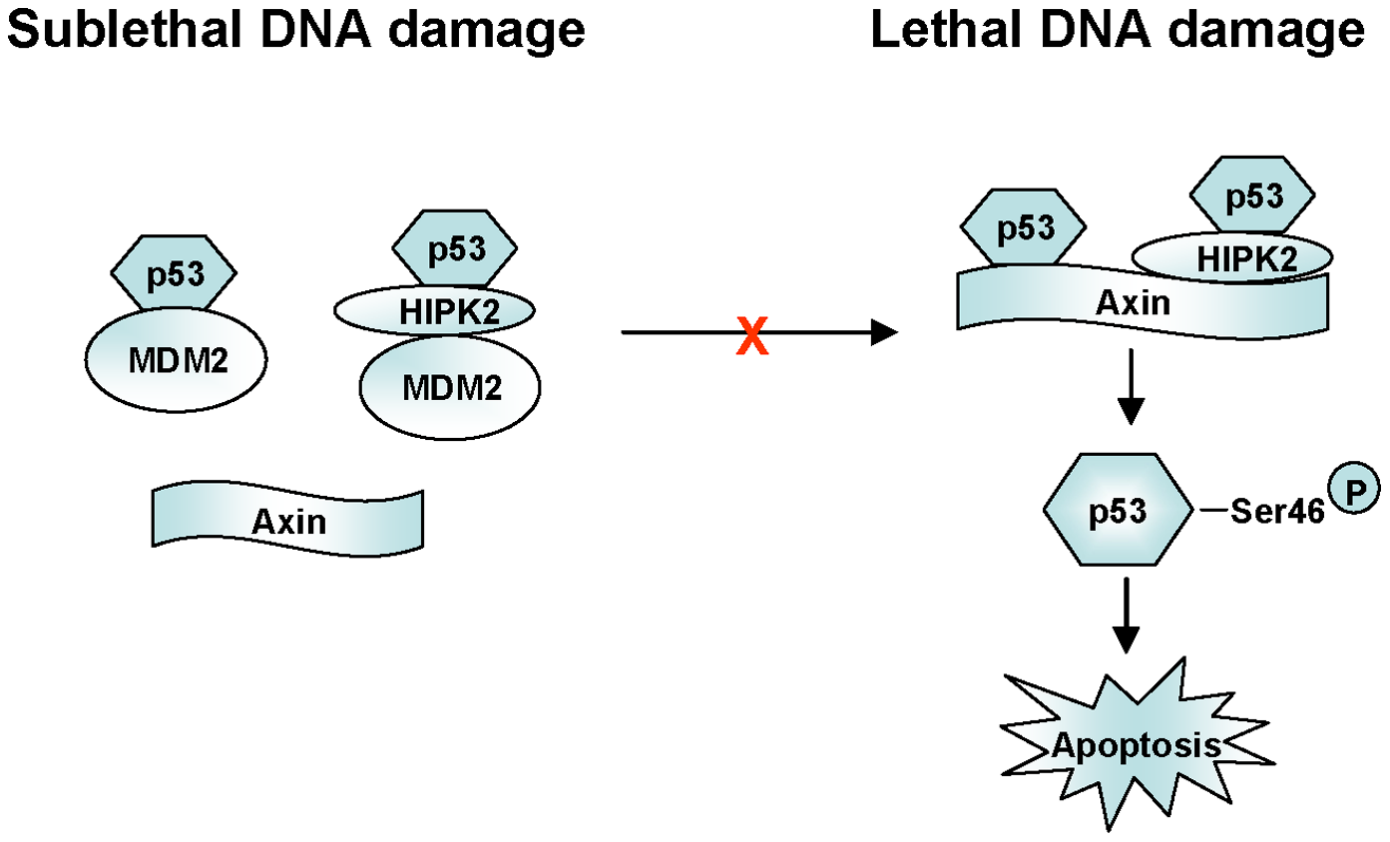
A simplified model depicting the mechanism how MDM2 inhibits Axin-induced p53 activation. When overexpressed, MDM2 competes the binding of both p53 and HIPK2 on Axin, and disrupts the Axin/p53/HIPK2 complex, leading to the inhibition of p53 activation and cell apoptosis.

It is well established that the principal action of MDM2 in p53 down-regulation is to poly-ubiquitinate p53, leading to proteasomal degradation of p53 [1], [2]. Our data showed that the E3 ligase activity of MDM2 is not necessary to attenuate Axin-induced p53 activation. Although MDM2 does not interact with Axin directly (Figure 4B and Figure 5B), it can compete against Axin to bind p53 and HIPK2, then consequently detach the Axin/p53/HIPK2 complex. The binding domain of Axin and MDM2 on p53 is quite different, with the Axin binding domain at aa 36–290 [8], and the MDM2 binding domain at aa 14–27 or aa 40–54 [16], [17]. The competition may be caused by the protein conformational change or the different binding affinity. As MDM2 would degrade p53 in the cells, after transfection of MDM2 and its E3 ligase-dead mutant MDM2 (C464A) in each competitive experiment, we used the proteasome inhibitor MG132 to make the basal level of p53 equal, then observed the competitive binding. But the problem is that the protein levels in *vivo* are changing all the time, especially under genotoxic stress. It is a very complex dynamic process. We propose a simple model of MDM2 inhibit Axin-induced p53 transcription activation (Figure 6). When cells were under non-severe DNA damage, p53 was activated and stimulated the expression of MDM2. High level MDM2 could detach Axin/p53/HIPK2 complex by disrupting the Axin/p53 and Axin/HIPK2 interaction separately, then inhibited the activation of p53 Ser 46 phosphorylation which is considered to drive cells to undergo apoptosis, and eventually protect cells from apoptosis. When cells were under severe irreversible DNA damage stress, MDM2 induction was absent [18] and MDM2 levels were not enough to inhibit the formation of Axin/p53/HIPK2 complex. Axin serves as a scaffold to tether HIPK2 and p53 together [8], enhance the phosphorylation of p53 at Ser 46, and increase the transcriptional activity of p53, leading cell to udergo apoptosis. Our data show that MDM2 may function in the same way like another E3 ligase Pirh2 in Axin-p53 pathway [15]. It has been shown that in normal or sublethally damaged cells the Axin-p53 complex is primarily occupied by Pirh2 to avoid activation of HIPK2 kinase activity; In contrast, upon lethal damage, Tip60 is activated by ATM/ATR and subsequently binds to Axin, leading to dissociation of Pirh2 from Axin and allowing the formation of a complex composed of Axin, HIPK2, p53 and Tip60 that leads to maximal activation of p53 through phosphorylation at Ser 46, which ultimately triggers apoptosis [15]. It is important to note that death domain-associated protein Daxx is also involved in Axin/p53/HIPK2 complex formation [9]. Daxx cannot directly interact with p53, but is tethered to p53 complex using Axin as a bridge [19]. The formation of Axin/p53/HIPK2/Daxx complex leads to maximal stimulation of p53 activation [9]. In addition, MDM2 was shown to be capable of interacting with both Tip60 and Daxx, and subsequently mediating the ubiquitination of them [20], [21]. Taken together, MDM2 may play multifaceted roles in regulating p53 signals. Many details relating to its delicate regulatory mechanisms are still awaiting to be investigated.

## Supporting Information

Figure S1 **Wild type MDM2 shows strong E3 activity toward p53.** H1299 cells were transfected with 0.1 µg of HA-p53 and 0.8 µg of FLAG-Ubiquitin, together with 2 µg of Myc-MDM2 or Myc-MDM2 (C464A). 24 h posttransfection, cells were treated with 10 µM of proteosome inhibitor MG132 for another 4 h, followed by immunoprecipitation with HA antibody for p53. Immunoprecipitates were detected with FLAG, HA and Myc for ubiquitin, p53 and MDM2 individually.
(TIF)

Figure S2 **Knockdown of** ***Mdm2*** **increases the interaction of Axin and p53.** U2OS cells were infected with lentivirus produced from pLL3.7 vector carrying control siRNA or siRNA against *Mdm2*. After 24 hours of infection, cells were selected with 1 mg/ml of neomycin for 10 days followed by immunoprecipitation and western blotting to detect p53, MDM2 and Axin.
(TIF)
