# Supplementary figures and images for: MDM2 Inhibits Axin-Induced p53 Activation Independently of its E3 Ligase Activity
Source: PLoS One. 2013 Jun 27;8(6):e67529. doi: 10.1371/journal.pone.0067529 (PMC3694902; doi:10.1371/journal.pone.0067529)

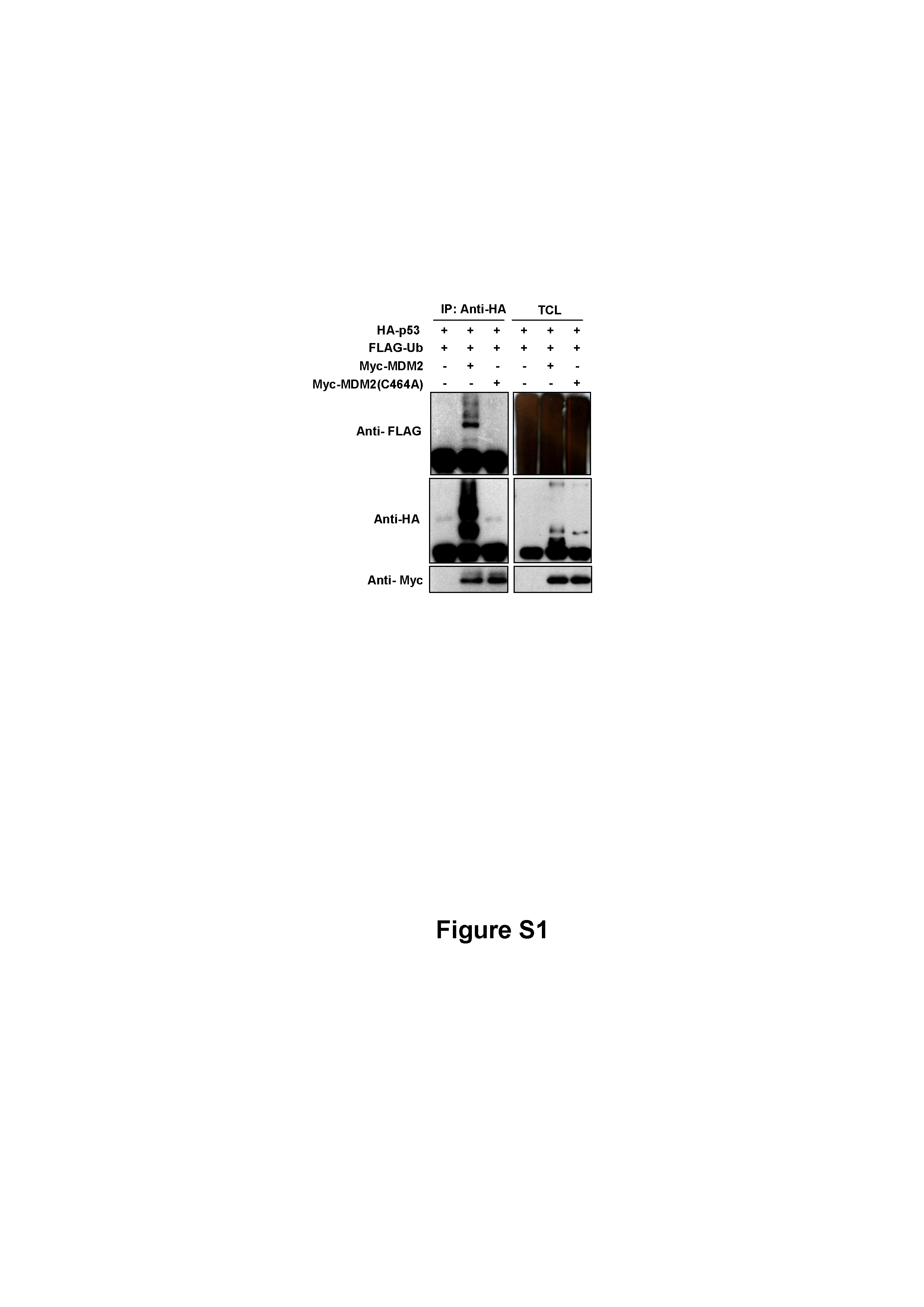

Supplement: Figure S1 — Wild type MDM2 shows strong E3 activity toward p53. H1299 cells were transfected with 0.1 µg of HA-p53 and 0.8 µg of FLAG-Ubiquitin, together with 2 µg of Myc-MDM2 or Myc-MDM2 (C464A). 24 h posttransfection, cells were treated with 10 µM of proteosome inhibitor MG132 for another 4 h, followed by immunoprecipitation with HA antibody for p53. Immunoprecipitates were detected with FLAG, HA and Myc for ubiquitin, p53 and MDM2 individually. (TIF) [file pone.0067529.s001.tif]

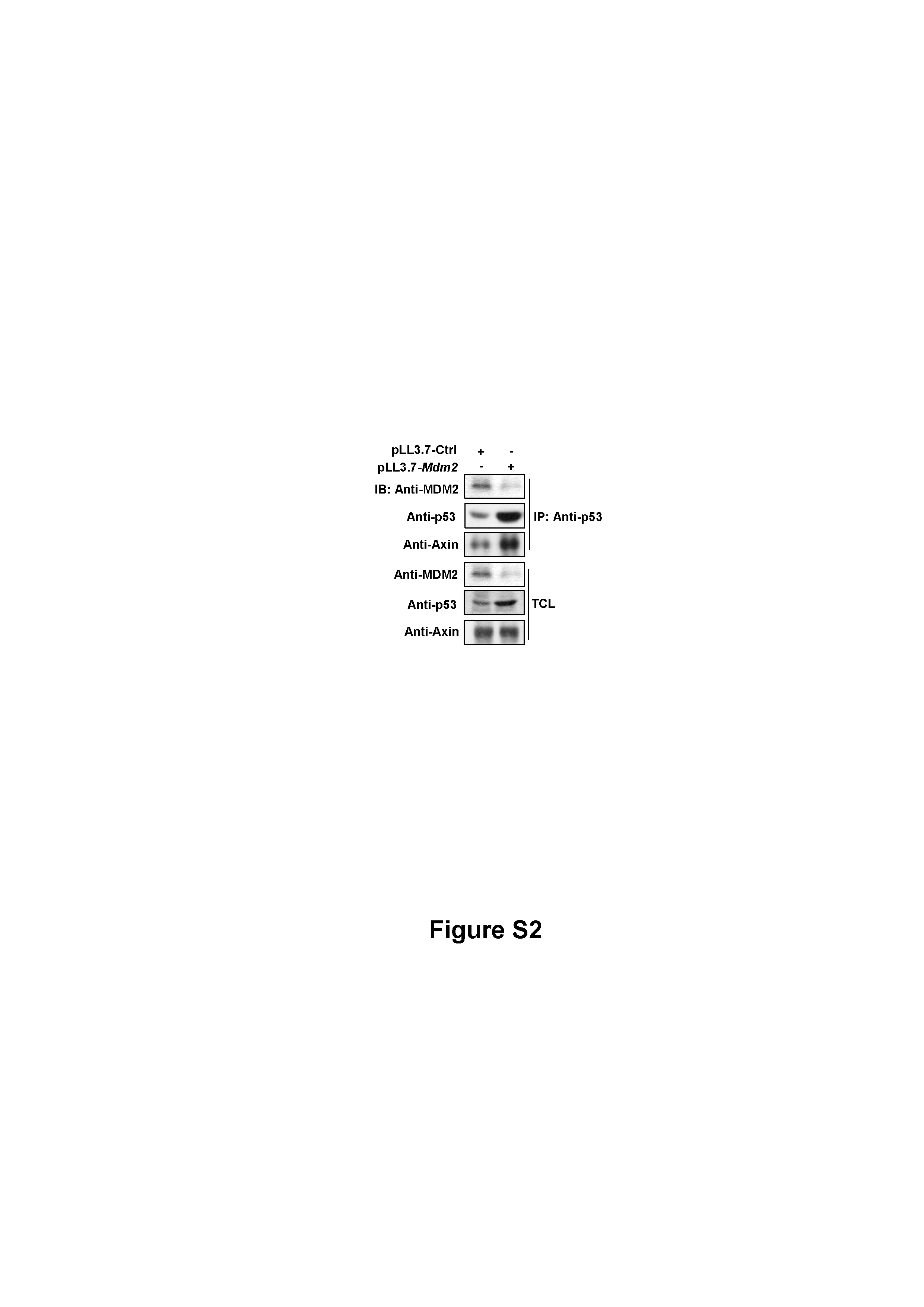

Supplement: Figure S2 — Knockdown of Mdm2 increases the interaction of Axin and p53. U2OS cells were infected with lentivirus produced from pLL3.7 vector carrying control siRNA or siRNA against Mdm2. After 24 hours of infection, cells were selected with 1 mg/ml of neomycin for 10 days followed by immunoprecipitation and western blotting to detect p53, MDM2 and Axin. (TIF) [file pone.0067529.s002.tif]
